# Use of digital media by adolescents for sexual and reproductive health and rights communication in sub-Saharan Africa: a protocol for systematic review

**DOI:** 10.1186/s13643-024-02534-z

**Published:** 2024-05-14

**Authors:** Josephine Akua Ackah, Kobina Esia-Donkoh, Antoinette P. Amponsah, Wonder Agbemavi, Vincent Bio Bediako, Gloria N. A. Tettey

**Affiliations:** 1 Department of Population and Health, Faculty of Social Sciences, College of Humanities and Legal Studies, University of Cape Coast, Cape Coast, Central Region Ghana; 2 Marie Stopes International, Accra, Ghana

**Keywords:** Digital media, Adolescents, Sexual and reproductive health and rights, Sub-Saharan Africa

## Abstract

**Background:**

Within the sub-Saharan African region, there is a growing concern for sexual and reproductive health and rights communication, and more particularly, for adolescents. Given the existing barriers associated with face-to-face access, the need to use digital media to access information and services has become desirable and imperative, especially so due to the COVID-19 pandemic. However, in sub-Saharan Africa, a synthesis of evidence that informs adolescents’ digital media engagements for sexual and reproductive health and rights (SRHR) communication is limited. This systematic review therefore aims to examine and synthesize evidence on use of digital media for sexual and reproductive health and rights communication by adolescents in sub-Saharan Africa.

**Methods:**

A search for peer-reviewed articles will be conducted in PubMed, ScienceDirect, Scopus, Embase, Web of Science, PsychINFO and Google Scholar with emphasis on those published between 2000 and 2023. Only observational and qualitative studies will be included. Quality assessment of included articles will be done using standardized checklists from the Joanna Briggs Institute. Both descriptive and narrative summaries will be used to appraise evidence from included studies.

**Discussion:**

This review will be essential in providing information on the types of digital media adolescents use, the various SRHR issues they use this platform to address and their reasons for using it and associated challenges. It will also contribute to the advocacy for the inclusion of these technologies in the teaching and learning, provision of and access to SRHR information and services by teachers, public health providers and peer educators in the subregion.

**Systematic review registration:**

PROSPERO CRD42020211491. This protocol follows the PRISMA-P guidelines for reporting systematic reviews.

**Supplementary Information:**

The online version contains supplementary material available at 10.1186/s13643-024-02534-z.

## Introduction

Meeting the sexual and reproductive health and rights (SRHR) needs of adolescents is one of the major concerns of governments and institutions around the globe, and especially, in Africa [1–4]. In developing nations, access to information, contraceptive usage and ability to make informed sexual decision and practice safe sexual life are hindered by several barriers such as financial constraints, cultural limitations, misconceptions, limited access to adolescent-friendly services and stigma and discrimination [5, 6]. These barriers are often founded on both structural and sociocultural norms/expectations that shroud sexual issues in secrecy, making it particularly challenging for groups like adolescents to harness available resources [7].

Sexuality education is a prominent tool that several governments in sub-Saharan Africa have adopted to reach adolescents with information and services on SRHR, in line with national and international directives [3, 4]. The transition has moved from public education to comprehensive sexuality education targeting both in-school and out-of-school adolescents while integrating several stakeholders such as teachers, parents and health providers [8]. Nevertheless, concerns on privacy, stigma and discrimination as well as inadequate delivery of information and services are but a few challenges this mode of communication is saddled with [1, 9]. Furthermore, in the face of emergencies such as the COVID-19 pandemic, face-to-face strategies tend to be ineffective due to restrictions (including lockdowns, curfews and other safety protocols) [10]. The need, therefore, to explore other effective approaches which promote privacy and reduce stigma becomes not only imperative but also timely.

In the twenty-first century, digital technologies have become a necessity and possess the power needed to offset the challenges associated with SRHR face-to-face communication strategies [11]. In the Maputo Plan of Action, for instance, advocacy for the use of new communication technologies has been emphasized [3]. Moreover, with the continual increase in the possession of mobile phones and other communication devices among the youth [12], integrating digital technologies in SRHR will incense rippling advantages.

Existing systematic reviews that explore the use of digital media have been conducted but largely in European contexts including studies by Guse et al. [11] and Wadham et al. [13]. Additionally, in a systematic review protocol by Feroz et al. [14], focus is primarily on only adolescents’ use and engagement in mHealth interventions. These reviews offer a narrow perspective on the experiences of adolescents in the African settings [11, 13, 14]. This review will expansively investigate how digital technology has been used by adolescents in sub-Saharan Africa to access information about their sexuality, reproductive health and rights. The ultimate edge of this review is to establish the basis for incorporating digital technologies in the teaching and learning, provision of and access to information and services on SRHR to adolescents by teachers, public health providers and peer educators in sub-Saharan Africa.

### Objectives

In the systematic review, we aim to identify, critically synthesize and undertake a systematic review on how digital media are used by adolescents in sub-Saharan Africa to access SRHR information and services. Specifically, we will assess/rationalize the following:

1. Types of digital media that adolescents use to access SRHR information and services and the specific SRHR issues they use digital media for
2. Reasons for using digital media and challenges/limitations associated with it
3. Adolescents’ experience with access to SRHR information and services using digital media (availability, affordability, appropriateness and acceptability)

## Methods

### Eligibility criteria

#### Type of studies

We will include all published observational studies to investigate the usage of digital media by adolescents in sub-Saharan Africa for SRHR purposes. In addition to these, qualitative studies (including those from mixed-methods studies) and other grey literature that explore the study interests will be included. However, reviews, comments, randomized control trials, clinical trials and case studies will be excluded.

#### Type of participants

The participants for this study are adolescents (10–24 years). According to the World Health Organization [15], the age category for individuals who are considered adolescents is 10–19 years. However, in this review, we will extend this to those aged 20–24 years since some individuals experience late adolescence [16] and have similar SRHR experiences as those in the 10–19-year age group [17].

#### Setting

We will include studies that have been conducted in countries in sub-Saharan Africa. These countries include Angola, Benin, Botswana, Burkina Faso, Burundi, Cameroon, Central African Republic, Chad, Congo, Cote d'Ivoire, Eritrea, Ethiopia, Gabon, Gambia, Ghana, Guinea, Guinea-Bissau, Kenya, Lesotho, Liberia, Madagascar, Malawi, Mali, Mauritania, Mauritius, Mozambique, Namibia, Niger, Nigeria, Rwanda, Senegal, Sierra Leone, Somalia, South Africa, United Republic of Tanzania, Togo, Uganda, Zaire, Zambia and Zimbabwe.

#### Type of exposure

Only studies that report on the use of digital media will be included. Digital media encompasses social networking sites (social media), podcasts, vodcasts and text messaging. Studies to be included must link the use of digital media to sexual and reproductive health and rights issues.

#### Information sources

A search for published peer-reviewed articles on digital media and its use for SRHR by adolescents in sub-Saharan Africa will be performed in the following databases: PubMed, ScienceDirect, Scopus, Embase, PsychINFO, Web of Science and Google Scholar. Studies will be included if they were published from 2000 to 2023. Search strategies that employ Medical Subject Headings (MeSH) will be used in identifying studies to be included. The reference list of included studies will be screened to identify additional potential studies that might be useful. We will also contact experts in the field for additional peer-reviewed documents that will be essential for this review. Only studies published in English will be considered since all authors are proficient in this language.

#### Search strategy

Specific search strategies will be developed by two authors, and afterwards, a librarian at the University of Cape Coast Library Services will be consulted for confirmation. This will further be reviewed by the remaining authors and concluded upon. Medical Subject Headings pertinent to each of the databases will be used in the search. In general, the following search terms will be considered: “digital media”, “social media”, “text messaging”, “internet”, sex, sexual, reproductive, “sexual and reproductive health”, “reproductive health”, “reproductive rights”, types, kinds, challenges, reasons, experiences, access, affordability, availability, acceptability, appropriateness, etc. Boolean operators such as “AND” and “OR” will be used to define how search terms will be combined. Search strategy that will be used in PubMed will be adapted for other databases (see Additional file 2).

### Study records

#### Data management

Studies that will be retrieved from the database search will be managed using DistillerSR. DistillerSR is a literature review software that efficiently employs the strengths of artificial intelligence (AI) in automating literature collection, screening and assessment. Furthermore, it has in-built tools that facilitate effective collaboration among reviewers on decisions to include or exclude articles. Justification for excluding studies will be provided by reviewers (J. A. A., W. A., G. T.) and later discussed to be approved by the group. To ensure consistency, the reviewers will be guided by the eligibility criteria stated above to facilitate the selection process, and calibration will be carried out before starting our review.

#### Selection process

At least, three review authors (J. A. A., W. A., G. T.) will independently screen titles and abstracts against the inclusion criteria. Full reports of all titles that meet the inclusion criteria will be obtained. Where there are uncertainties, an additional reviewer (K. E.) will arbitrate and drive a discussion to arrive at desired conclusions.

#### Data collection and items

A standardized data extraction form will be designed to collect the needed information from included articles. Two reviewers (A. P. A., V. B. B.) will spearhead the data collection process. Information to be included is as follows: article information (authors, date, title, journal, publisher, funding, etc.), population characteristics (age groups of participants, sex), study setting, study design, type of digital media, SRHR issues, reasons for using digital media, challenges, projects and any other useful information. A third reviewer (K. E.) will address any dissensions.

#### Outcomes

In this review, we aim at addressing five crucial issues on the use of digital media by adolescents in sub-Saharan Africa. They include (1) types of digital media that adolescents use for SRHR communication, (2) reasons for using digital media, (3) specific SRHR issues that adolescents use digital media to access, (4) the limitations associated with using digital media and (5) their experience with accessing digital media: specifically, availability, affordability, acceptability and appropriateness.

### Quality assessment

The quality of included studies will be assessed using standardized checklist tools designed by the Joanna Briggs Institute (JBI). They can be accessed at https://jbi.global/critical-appraisal-tools. JBI has 13 different study-specific checklists to assess their methodological quality such as the design, conduct and analysis of respective studies. The JBI checklists will be chosen because they are standardized and suitable for the appraisal and assessment of most types of studies [18]. For this review, five checklists will be explored — checklists for analytical cross-sectional studies, case–control studies, cohort, prevalence and qualitative studies. Generally, the areas that are covered in the checklists for quality appraisal include sampling, measurement of exposure and outcomes, completeness and aptness of statistical analysis [19–21]. The quality assessment will be done by four reviewers (J. A. A., K. E., A. P. A., G. T.).

### Data synthesis

Both descriptive and narrative summaries will be used to appraise evidence from the sampled studies. We will provide frequencies of the types of digital media adolescents in sub-Saharan Africa use as well as the SRHR issues they use these media to access. We will use graphs and tables to illustrate our descriptive results. For the narrative summary, we will adopt a thematic analysis approach to identify similar and contrasting themes that run across the included studies [22, 23]. Findings from this review will centre around four main themes: types of digital media, reasons for using digital media to access SRHR issues, specific SRHR issues and limitations associated with using digital media and accessibility (e.g. affordability and availability) associated with digital media use. All authors will be involved in the data synthesis of included papers.

## Discussion

This review is important in updating the academic community and decision-makers on the various digital media platforms that adolescents are currently using to communicate issues on SRHR, with particular interest in experiences from sub-Saharan Africa. It will help decision-makers in their quest to identify common platforms adolescent use in order to streamline them into their e-strategical project and planning to enhance adolescent sexual and reproductive health. The findings from this study will also provide comprehensive information on the top SRHR issues that adolescents seek to address in the subregion. Additionally, a catalogue of the challenges associated with the use of digital media will help information providers in considering the risks involved in the use of digital media and how it can be minimized to ensure smooth delivery of SRHR information to adolescents. We hope that the findings from this study will propel the need for institutionalized inclusion of digital technologies in the teaching and learning, provision of and access to SRHR information and services by teachers, public health providers and peer educators.

### Supplementary Information

**Additional file 1.** PRISMA-P 2015 Checklist.

**Additional file 2. **Search Strategy for Pubmed

## Data Availability

Not applicable.

## Abbreviations

SRH: Sexual and reproductive health
SRHR: Sexual and reproductive health and rights
SSA: Sub-Saharan Africa
