# Supplementary material for: Use of digital media by adolescents for sexual and reproductive health and rights communication in sub-Saharan Africa: a protocol for systematic review
Source: Syst Rev. 2024 May 14;13:130. doi: 10.1186/s13643-024-02534-z (PMC11092230; doi:10.1186/s13643-024-02534-z)
Supplement: Supplementary file 2 — Additional file 2. Search Strategy for Pubmed [file 13643_2024_2534_MOESM2_ESM.docx]

**ADDITIONAL FILE 2**

**SEARCH STRATEGY FOR PUBMED**

**BLOCK 1**: “Digital media”[tiab] OR “new media” [tiab] OR “new technolog*” [tiab] OR "Social Media" [MeSH] OR “social media” [tiab] OR "Text messaging"[MeSH] OR “text messaging” [tiab] OR "social networking" [MeSH] OR “social networking site*” [tiab] OR internet [tiab] OR Webcast* [MeSH] OR Facebook [tiab] OR WhatsApp [tiab] OR Game* [MeSH] OR Game* [tiab]

AND

**BLOCK 2**: Adolescen* [MeSH] OR adolescen* [tiab] OR "Young people"[tiab]

AND

**BLOCK 3**: Sex [MeSH] OR sex [tiab] OR sexuality [MeSH] OR sexuality [tiab] OR "Reproductive Health" [MeSH] OR “reproductive health” [tiab] OR “rights, reproductive” [tiab] OR "sexual rights" [tiab] OR contraceptive* [MeSH] OR contraceptive*[tiab] OR Abortion [MeSH] OR abortion [tiab]

AND

**BLOCK 4**: Africa OR sub-Saharan Africa OR Angola OR Benin OR Botswana OR Burkina Faso OR Burundi OR Cameroon OR Central African Republic OR Chad OR Congo OR Cote d'Ivoire OR Eritrea OR Ethiopia OR Gabon OR Gambia OR Ghana OR Guinea OR Guinea-Bissau OR Kenya OR Lesotho OR Liberia OR Madagascar OR Malawi OR Mali OR Mauritania OR Mauritius OR Mozambique OR Namibia OR Niger OR Nigeria OR Rwanda OR Senegal OR Sierra Leone OR Somalia OR South Africa OR United Republic of Tanzania OR Togo OR Uganda OR Zaire OR Zambia OR Zimbabwe

AND

**BLOCK 5:**

Reason OR challenge* OR experience OR access OR afford* OR availab* OR limit*
